# Understanding Cervical Screening Engagement Among Young South Asian Women: Perspectives of Women and Healthcare Professionals

**DOI:** 10.1111/hex.70960

**Published:** 2026-11-05

**Authors:** Katie Merry, Zainab Laheri, Sarah Johns, Yasemin Hirst, Rebecca Jefferson

**Affiliations:** ^1^ Lancashire and South Cumbria NHS Foundation Trust, Trust HQ, Sceptre Point, Sceptre Way, Walton Summit Preston UK; ^2^ IMPlementation and Capacity Building Team (IMPaCT), Health Technology Assessment (HTA) Unit, Applied Health Research hub (AHRh), University of Central Lancashire Preston Lancashire UK; ^3^ Healthwatch Blackburn with Darwen, Unit 19 Eanam Wharf Business Centre Blackburn UK

**Keywords:** cervical screening, health inequalities, primary health care, South Asian Women

## Abstract

**Background:**

Despite increased uptake of the human papillomavirus (HPV) vaccine, cervical screening participation among younger South Asian women has declined, particularly in deprived areas in England. The reasons for this pattern remain poorly understood. This study explored barriers and facilitators to cervical screening among South Asian women aged 25–29 in a deprived locality.

**Methods:**

Semi‐structured interviews were conducted with South Asian women aged 25–29 (*n* = 15) and healthcare professionals involved in cervical screening delivery (*n* = 6) in Blackburn with Darwen. Data were analysed using reflexive thematic analysis.

**Results:**

Barriers to screening were multifactorial and included limited understanding of cervical screening and HPV, cultural norms surrounding modesty and sexuality, fear of community judgement and embarrassment, and structural challenges such as appointment access and competing responsibilities. Family networks acted as both barriers and facilitators. Healthcare professionals described adapting language to reduce fear, but highlighted service pressures related to staffing and funding, which constrained proactive engagement. Although most women reported receiving the HPV vaccination, understanding of HPV and the continued importance of cervical screening remained limited, highlighting opportunities to reinforce cervical screening education.

**Conclusion:**

Cervical screening engagement among young South Asian women is shaped by intersecting cultural, emotional, and structural influences, which may be intensified in deprived settings. HPV vaccination should be accompanied by clear communication about the continued importance of cervical screening, particularly where knowledge and awareness remain low. Culturally responsive communication, flexible service provision and community partnership approaches are needed to address persistent inequalities in screening uptake.

**Patient or Public Contribution:**

The study was informed by prior community engagement and public involvement work with underserved populations. Interview guides were developed in collaboration with a public advisor from the South Asian community to ensure they were relevant, inclusive, and reflective of community priorities.

## 1 Introduction

Cervical cancer remains an important public health issue worldwide and in the United Kingdom. Global and national strategies aim to eliminate cervical cancer through the human papillomavirus (HPV) vaccination at a younger age, and through screening and treatment of pre‐cancerous cells. In England, the National Health Service (NHS) has pledged to eliminate cervical cancer by 2040 [1], a target that could save thousands of lives each year. Preventive efforts include the routine HPV vaccination offered to girls and boys aged 12–13 since 2008, aimed at reducing transmission of high‐risk HPV types [2], and the NHS Cervical Screening Programme, which invites women aged 25–64 every 5 years since 1988 to detect and prevent cervical cancer at an earlier stage [3]. Evidence demonstrates significant reduction in cervical cancer incidence among those who are vaccinated and subsequently screened, highlighting the importance of uptake of both vaccination and screening programmes [4].

Despite historically high national coverage, cervical screening participation in England has declined steadily since 2010. Among women aged 25–49, coverage has fallen from 74.1% to 68.8% in 2024, with disparities across communities, in particular those from ethnic minority backgrounds [5]. These inequalities also extend to the HPV vaccination programme [6] with strong evidence indicating that those who are HPV vaccinated at age 12–13 are also more likely to attend cervical screening when invited for the first time [7, 8]. Despite this, less is known about how HPV vaccination relates to cervical screening engagement among underserved and minority ethnic groups. Research from England suggests that differences in vaccination and screening uptake by ethnicity may contribute to inequalities in HPV‐related disease, highlighting the importance of considering vaccination alongside continued participation in cervical screening [9]. However, this population‐level data does not capture individual‐level factors that may drive disengagement from screening after vaccination. Vaccination alone may therefore not be sufficient to support sustained engagement with cervical screening, particularly where knowledge, cultural, emotional or structural barriers persist.

The cervical screening inequalities literature consistently reports lower screening participation among South Asian women globally, particularly among younger cohorts, who may encounter distinct or intensified barriers to screening [5, 10, 11, 12, 13, 14, 15, 16]. Specifically, research highlights South Asian women often report limited knowledge of cervical cancer and screening, misconceptions related to risk of screening and emotional barriers including embarrassment, fear, and anticipated stigma. Cultural norms relating to modesty, language barriers, and difficulties navigating or engaging with health services are also frequently documented [10, 13, 15, 16]. However, much of this evidence is derived from studies encompassing heterogeneous ethnic groups and wide age ranges, limiting insight into the experiences of younger South Asian women approaching cervical screening for the first time. Moreover, there remains a limited understanding of how HPV vaccination delivered in adolescence intersects with subsequent cervical screening behaviour within this population.

Importantly, little qualitative research has examined cervical screening behaviour among South Asian women aged 25–29, many of whom are likely to have been eligible for HPV vaccination during adolescence. Although the first routinely vaccinated cohorts became eligible for cervical screening in England from 2020, there remains a notable absence of research exploring how HPV vaccination is understood, interpreted and contextualised within this population, and whether it meaningfully influences subsequent screening behaviour. Understanding this intersection is critical, particularly given the potential for HPV vaccination to shape perceptions of cervical cancer risk and the perceived necessity of continued screening.

In England, NHS data do not routinely capture cervical screening coverage by ethnicity, limiting detailed population‐level analysis of inequalities in screening participation. As a result, a place‐based approach is necessary to understand how inequalities in cervical screening participation are experienced within South Asian communities and how local, social, cultural, and structural factors may shape engagement. In the North West of England, Blackburn with Darwen represents a particularly pertinent setting, given the area's well‐documented health inequalities and sizeable South Asian population (over 35% of the residents identify as South Asian [17];). Cervical screening coverage in this locality remains below the national average, with coverage reported as 59.6% among women aged 25%–49% and 70.6% among women aged 50–64 in 2023 [18]. Examining screening participation and engagement within this context provides an opportunity to explore how broader patterns of inequality manifest at community level and to identify specific gaps in provision and access. Accordingly, this study aims to explore how cultural, social, structural and knowledge‐related factors influence cervical screening participation and engagement among South Asian women aged 25–29 living in Blackburn with Darwen.

Specifically, the study addresses the following research questions:

1. What are the perceived barriers and facilitators to cervical screening participation among South Asian women aged 25–29?
2. How do cultural, social and structural factors influence screening behaviour within this population?
3. How do healthcare professionals perceive the challenges and opportunities in supporting screening uptake?

## 2 Methods

### 2.1 Study Design

A qualitative study design was used to explore awareness, beliefs, and experiences related to cervical screening among South Asian women, alongside healthcare professionals' perspectives on screening delivery. A qualitative approach was considered appropriate as it enabled in‐depth exploration of participants' experiences, perceptions, and decision‐making processes surrounding cervical screening behaviour. The study is reported in accordance with the Consolidated Criteria for Reporting Qualitative Research (COREQ) checklist [19], provided as Supporting Information A.

### 2.2 Study Setting

This study was conducted in Blackburn with Darwen, a local authority area in the North West of England characterised by a large South Asian population and persistently low cervical screening coverage. This setting was selected to enable an in‐depth, place‐based examination of cervical screening inequalities within a community that experiences multiple forms of disadvantage.

### 2.3 Selection and Description of Participants

Two participant groups were recruited. The first group comprised of South Asian women aged 25–29 years living in Blackburn with Darwen who were eligible for cervical screening. Women who had undergone a total hysterectomy (including removal of the cervix) or who had formally opted out of cervical screening were excluded. This group of participants was recruited using opportunistic and snowball sampling through community networks, social media, local organisations, and professional contacts. Previous evidence suggests that developing interpersonal relationships with South Asian communities and building trust are integral to recruiting research participants [20]. Thus, lead researcher, KM, who worked at a patient advocacy organisation (Healthwatch Blackburn with Darwen), carried out patient and public engagement (PPIE) before recruitment with South Asian women aged 25–29 to identify best places for sharing recruitment materials and attended family hubs (i.e. organisations which provide support for women) to disseminate study information more widely. Where possible, if individuals were contacted to take part in the study, they were asked to share the study invitation wider in their communities. This approach was considered appropriate for accessing populations that are often under‐represented in health research and may be difficult to engage through traditional recruitment approaches.

The second group included healthcare professionals working in Blackburn with Darwen who had experience delivering cervical screening or women's health services. Eligible healthcare professionals included registered nurses, nurse practitioners, physician associates, doctors and practice managers. Healthcare professionals were recruited through existing professional and community networks, including Healthwatch Blackburn with Darwen and the Blackburn with Darwen GP Federation. Recruitment information was disseminated via invitation emails, social media and study advertisements, with opportunistic and snowball sampling used to identify additional participants. As healthcare professionals were recruited from services operating within Blackburn with Darwen, where South Asian communities comprise a substantial proportion of the local population, participants would be expected to have routine experience providing care to South Asian patients. Consequently, specific information regarding previous experience working with South Asian communities was not collected.

Recruitment materials for both participant groups outlined the study purpose and eligibility criteria. All participants received a £20 shopping voucher in recognition of their time and contribution. No participants withdrew after providing consent, and reasons for non‐participation were not systematically recorded. Recruitment continued until sufficient information power was achieved, taking into consideration the specificity of the sample, the focused study aim and the locality in which the study was conducted [21].

### 2.4 Data Collection

Semi‐structured, one‐to‐one interviews were conducted between October 2024 and January 2025. Interviews took place either face‐to‐face, by telephone, or via Microsoft Teams, according to participant preference, and lasted approximately 30 min. No non‐participants were present during interviews. All interviews were audio‐recorded and transcribed verbatim.

Separate interview guides were developed for South Asian women and healthcare professionals. For women participants, questions explored knowledge and awareness of cervical screening, accessibility and logistical challenges, previous screening experiences, perceived barriers and facilitators to participation, and cultural or social influences on screening engagement. Healthcare professionals were asked about commonly reported patient concerns and misconceptions, service‐level barriers and facilitators, strategies used to support screening uptake and perceived cultural influences on participation.

To ensure the interview guides were relevant, inclusive, and reflective of community priorities, they were developed in collaboration with a public advisor from the South Asian community in Blackburn with Darwen. This collaborative approach helped to shape the wording and focus of the questions, supporting the collection of meaningful and culturally sensitive insights and strengthening the study's relevance to the population of interest. The interview schedules are provided in Supporting Information B and C.

### 2.5 Data Analysis

All interviews were conducted and transcribed verbatim by KM. Data were managed and coded in NVivo (v14.23.3) and analysed using reflexive thematic analysis, following Braun and Clarke's [22] six‐phase approach. Transcripts were read repeatedly to achieve familiarisation with the data and to identify initial patterns of meaning. Coding was conducted inductively, with codes generated directly from the data rather than through pre‐defined categories. The coding process was iterative and reflexive, involving active engagement with participants' accounts to identify patterns within and across interviews. Initial themes were developed through grouping related codes and were subsequently reviewed and refined to ensure they accurately represented the dataset. Themes were then defined and named through an iterative process of reflection and team discussion. Analysis was conducted by YH and ZL. Regular discussion within the wider research team supported analytical rigour by encouraging reflexive engagement, critical examination of assumptions, and consideration of alternative interpretations.

### 2.6 Researcher Characteristics and Reflexivity

The study was conducted by a multidisciplinary research team with experience in qualitative health research, public health, and cancer prevention. Team members all identified as female and brought a range of professional backgrounds and perspectives to the design, conduct, and analysis of the study. Data collection and analysis were undertaken by researchers trained in qualitative interviewing and thematic analysis. Reflexivity was maintained throughout the research process through regular analytic discussions, during which the team reflected on how their assumptions, disciplinary perspectives, and prior understanding of cervical screening and healthcare engagement might shape data collection, interpretation, and theme development. This collaborative and reflexive approach supported transparency, analytic rigour, and credibility of the findings.

### 2.7 Ethical Considerations

Ethical approval was obtained from the University of Lancashire Ethics' committee (ref: HEALTH 01169). Data were anonymised and stored securely, with access limited to the research team. Anonymised transcripts were shared with a public advisor to support interpretation of the findings. All participants provided informed written consent prior to participation in the interviews.

## 3 Results

### 3.1 Participant Characteristics

A total of 15 South Asian women participated. Participants identified as Indian, Pakistani, British Indian or British Pakistani. Most reported at least one prior experience with cervical screening (*n* = 10), although some described delayed participation (*n* = 2) or non‐attendance (*n* = 3). HPV vaccination status varied, with the majority reporting receiving the vaccination (*n* = 10), while a small number were unsure or unable to recall (*n* = 4) (Table 1). Although most women had attended screening on at least one occasion, all participants met the study's eligibility criteria and were recruited from a locality with persistently low cervical screening coverage. As described below, many participants recounted inconsistent engagement with the screening pathway over time, and their accounts offer insight into the factors that supported eventual attendance.

**Table 1:** Demographic information for South Asian Women participants.

Characteristic	*n* = 15
Age (mean, range)	Mean = 27, range = 25–29
Ethnicity	
Indian	3
Pakistani	4
British Indian	1
British Pakistani	7
Married	10
Children	6
Previous cervical screening	
Never attended	3
Attended once	8
Attended more than once	2
Delayed participation	2
HPV vaccinated	
Yes	10
No	1
Unsure/don't remember	4

Six health care professionals participated, including general practice nurses (*n* = 4), a practice manager (*n* = 1) and an operations manager (*n* = 1). Healthcare professionals represented both primary care and sexual health clinic settings involved in screening delivery (Table 2). All healthcare professionals worked in Blackburn with Darwen from GP practices with high South Asian representation and their roles included delivering cervical screening, contacting non‐responders to reengage in screening and supporting women in informed decision‐making.

**Table 2:** Demographic information for healthcare professional participants.

Characteristic	*n* = 6
Place of work	
General practice	4
Sexual health service	2
Role	
General practice nurse	4
Practice manager	1
Clinical/operations manager	1

### 3.2 Qualitative Findings

Five key themes were developed following analysis of the interviews, illustrating how knowledge, structural realities, cultural context and emotional responses collectively shape cervical screening engagement among South Asian young women. A summary of each theme with an illustrative quotation has been provided in Table 3.

1. **Knowledge gaps and misconceptions**
  South Asian women revealed persistent uncertainty about the purpose and process of cervical screening, with many participants describing limited understanding of what screening involved or tested for. One participant mentioned, ‘I'll be very honest, I actually have no clue… I don't really know what it is or what it's looking for' (Patient, 1). This lack of clarity contributed to anxiety and shaped expectations of the procedure and informed responses to the invitations to screen.
  Misconceptions were frequently reinforced through imagined scenarios and informal information sources. Anticipated invasiveness was highlighted by one participant who described, ‘I'm thinking of like big metal forceps… like they're going to open you up. That's what I imagined.' (Patient, 4). HCPs similarly recognised widespread misunderstanding, particularly around HPV literacy, ‘I never refer to it as a cancer test, because if you're HPV positive it doesn't mean you've got cancer, but that's what a lot of women think.' (HCP 3)
  Knowledge gaps were situated within missed opportunities for education across the life course. Several women described little exposure to screening information in educational or healthcare settings, with one participant noting, ‘It wasn't talked about in school, neither college… it was never explained.' (Patient, 9). Participants frequently expressed uncertainty in their knowledge about the vaccine itself and its link to cervical screening. This uncertainty persisted even among participants who reported receiving the HPV vaccination, highlighting the importance of communicating that HPV vaccination forms part of cervical cancer prevention. Many participants had limited or no recall of what they'd received and why, with one participant mentioning ‘I don't think there's a lot about this vaccine online because I'm not going to lie to you after high school I still haven't even heard of it. I was trying to google it for days.' (Patient 10). Cultural factors also shaped vaccine participation, with one participant voicing how non‐attendance was common among peers, ‘A lot of people never had it because they thought it was Haram (forbidden).' (Patient 4). Similarly, HPC's observed that HPV knowledge was poorly understood among patients, ‘I think a lot of women… they don't understand HPV, they've never heard of it, so they don't actually know what's being tested… and it's because there's not a lot of education out there… about HPV and what it is and how we catch it.' (HCP, 6). Despite the majority of participants self‐reported receiving the HPV vaccination, this did not appear to translate into consistent engagement with cervical screening. Many women remained unclear about HPV and its relationship to cervical cancer and screening.
**Structural, practical and place‐based barriers to access**
  Practical constraints limited women's ability to attend screening. Difficulties navigating appointment systems were commonly described, with one participant stating, ‘Just ringing the doctors to book the appointment was a mission in itself.' (Patient, 2). Inflexible clinic schedules further compounded these challenges, especially for those women balancing work and caregiving responsibilities, ‘Work… family, kids and you're putting everyone else first all the time… we're too busy looking after everyone else all the time, aren't we.' (Patient, 6).
  On the other hand, participants further described how practical constraints relating to marriage and family could also influence screening behaviour. Some participants suggested being more open to screening after marriage, ‘I have had a screening recently. That was post marriage, so I had a screening after I was married.' (Patient 8), while for others screening was frequently deprioritised amid competing demands, as one participant explained, ‘I think maybe before I had kids, it'd probably be one of my top priorities.' (Patient, 11). This highlighted that marriage can act as a cultural prompt to increase screening behaviour, while the demands of family life could also introduce new practical barriers to consider. For some women who had previously delayed attending, marriage appeared to increase the perceived relevance of screening, suggesting a potential turning point at which openness to attendance grew.
  HCPs thought the financial and logistical burden associated with travel and parking reduced attendance, noting, ‘for somebody to come here for a cervical screen, it's you know, where we're going to park. Do I have enough money or for a taxi or whatever?' (HCP, 3). Moreover, even when services were geographically accessible, interviews suggested social visibility within local settings could create discomfort, with concerns about judgement or recognition creating barriers for attendance, with one HCP mentioning how ‘they don't want to be seen walking into a sexual health clinic…don't want people to think, oh, you know, why is she going in there?' (HCP 3).
  Furthermore, a common discussion was around the reminder letters and text messages sent by GP practices which were largely ineffective at prompting attendance among the participants. Several participants mentioned receiving multiple invitations without acting upon them, with one noting, ‘I would say I've probably received a letter maybe 3–4 times now… you don't find them that informative…' (Patient 1) and another participant stating, ‘I've been getting a letter, I got a letter again after that, but I've not done it.' (Patient 6). Letter content was frequently highlighted as a barrier itself, with participants describing them as inaccessible and uninformative, ‘not enough information about what it required or how it's going to be carried out, or what was the reason for it, it's that the lack of knowledge and the letter doesn't mention anything of that sort. So you just think, what am I signing up to?' (Patient 1). Some participants simply discarded the letter without reading them, ‘And me and my friends were like, oh, have you got it? Let's just put it in the dustbin.' (Patient 1). HCP's further acknowledged the limitations of written reminders and actively explored alternative approach to increase engagement, ‘I'm trying to increase the amount of text messages we're sending and making appointments bookable via the text because I think we are very much a techie world these days people don't want to sit on the phone for hours… got a chance of getting them in if they just click a link and say, yeah, I'll have that day and book it…(HCP, 4). One HCP noted the practical realities of forgetfulness which further compounded engagement, ‘By the time the appointment time has turned up… they tend to forget because it's 3 weeks in advance. So they've forgotten, you know, although they do get reminders.' (HCP, 5). These findings suggest that reminder systems alone are not sufficient, as non‐attendance is not only driven by difficulties with access, but additionally by gaps in understanding and broader cultural and structural barriers.
2. **Cultural and social contexts influencing engagement**
  Participants placed screening decisions within cultural norms around modesty, sexuality and reputation. For some women, screening before marriage conflicted with social expectations. One participant explained, ‘because the way we've all been brought up, well the majority of us you shouldn't really get it done before marriage.' (Patient, 8). Embarrassment around discussing intimate health was also a common feeling, with one participant describing, ‘I was whispering, can I make a smear test please?' (Patient, 11). Preferences for female practitioners were commonly expressed as a means of protecting comfort and dignity, ‘I'd definitely prefer a female. It's just more comfortable.' (Patient, 13).
  Family and community influences also played a significant role in shaping screening behaviour, operating as both facilitators and barriers. One participant described contrasting maternal influences, ‘my mum was very pro screening…but my friend's mum was like no, don't go for it. (Patient 10). Participants often relied on experiential knowledge among peers than medical information ‘Eight of my friends are scared to do it. I was the 9th, I've done and a few of them, they're considering it now. I said make sure you won't feel it.' This highlights how encouragement from family members and reassurance from peers who had already attended screening, appeared to help some women move from initial reluctance towards participation. One HCP noted that for married women the decisional influences also extend to their partners, ‘Some of them have to go back and get their husbands permission before they come back.' (HCP, 5). Trust and representation emerged as important facilitators of engagement. HCPs described improved uptake where language barriers did not exist, explaining, ‘I think the uptake has improved in the surgery, you know, because like I say, I can speak the language.' (HCP, 5). South Asian women highlighted the benefit of peer advocacy with one participant mentioning ‘I think people who have had it done, if they talk about it more within communities.' (Patient 13). Although cultural sensitivity remained important to consider, with one HCP noting ‘we have to be careful about where we use these videos. It's quite embarrassing in the Asian community.' (HCP, 5).
3. **Emotional drivers of engagement and avoidance**
  Fear and embarrassment were central to women's experiences of screening, and were often linked to limited knowledge and understanding of what screening involved. Anticipation around exposure was described, where women were uncertain about what to expect ‘I was like I have to get my bits out in front of another person. That's what I think I was more nervous about' (Patient, 10). For some, past healthcare experiences intensified this emotional burden, as illustrated by a participant who shared, ‘I had just given birth so I was very traumatised from giving birth so I said no.' (Patient, 12).
  Fear of results also shaped engagement. One participant described the distress associated with waiting for outcomes, ‘I was on edge, you know, for 8 days. I was like, oh yeah, cancer, what would I do? Yeah, you know what I'm saying? So very scary.' (Patient, 5). For some, avoidance also served as a coping mechanism, with another participant noting, ‘I just prevent myself from doing the test.' (Patient, 6). These responses suggest that for some women, the emotional burden associated with screening, including anticipation of the procedure and its findings, hindered participation in cervical screening. Despite these concerns, positive experiences also had the potential to reframe expectations. Reflecting on their screening experience, one participant mentioned, ‘And it's not as bad as you think it's going to be' (Patient, 4), a feeling echoed by HCPs who observed similar reactions following appointments. For women who had positive experiences, this kind of reassurance appeared to have played an important role in eventual attendance, indicating that emotional responses to screening could positively shift once initial engagement was achieved,
4. **Opportunities for change: community‐led and culturally responsive solutions**

**Table 3:** Summary of themes with illustrative quotations.

Subtheme	Illustrative quote
Theme 1: Knowledge gaps and misconceptions around cervical screening	
Low awareness of purpose and process	*‘I'll be very honest, I actually have no clue… I don't really know what it[cervical screening] is or what it's looking for’ (Patient 1; not screened, vaccinated)*
	*‘I'm thinking of like big metal forceps… like they're going to open you up. That's what I imagined.’ (Patient 4; not screened, vaccinated)*
	*‘I never refer to it as a cancer test, because if you're HPV positive it doesn't mean you've got cancer, but that's what a lot of women think.’ (HCP 3)*
	*‘They don't understand HPV. They've never heard of it, so they don't know what's actually being tested.’ (HCP 6)*
	*‘I think we had it just the girls because we were so excited that it's just the girls can get out of lesson. And when we were out of class. Yeah, all going together and we didn't know. We didn't know what was going in our arm.’ (Patient 9; screened, vaccinated)*
	*No, I don't think I know I've had one, but that was not quite sure what it [HPV vaccination] was. (Patient 2, screened, not vaccinated)*
	*‘don't know. I don't. I don't recall that. I don't think I did. I don't, don't. I don't know if that was. No. Is this something for like it's too far back.’ (Patient 14; screened, unsure if vaccinated)*
	*‘Yeah, I have heard of it [HPV vaccination], but I just can't remember if I had it done. And usually I think at that age it's more of the parents.’ (Patient 13; screened, unsure if vaccinated)*
*Misinformation and informal knowledge networks*	*‘I did google it and stuff…There's going to be mixed views on google, you don't know what's right or wrong’ (Patient 3; screened, vaccinated)*
	*‘A lot of people never had it [HPV vaccination] because they thought it was Haram.’ (Patient 4; not screened, vaccinated)*
	*‘I think some of the misconceptions is, is they've always heard a horror story or somebody's been into and had an experience. So I think with a lot of women, rather they listen to somebody else's story rather than experiencing it for themselves.’ (HCP 2)*
	*‘It is very much negatively reflected on. It is seen as why would you need that? Because you've not been sexually active. So if you're going for the test, then it must mean that you have been sexually active.’ (HCP 4)*
	*‘My sisters, the girls would be like, oh, I'm putting my kids for the forward for this vaccine. I don't know if it's for that, but. And then the brothers would stop People would be like, yeah, but it's got something in it gelatine you know, somewhere, I don't know.* *And then people that don't do it, but then some of them will do it and some of them won't then because scared.’ (Patient 4; not screened, vaccinated)*
*Missed educational opportunities across the life course*	*‘It wasn't told about in school, neither in college as well. It was never talked about.’ (Patient 9; screened, vaccinated)*
	*‘I would have to look into that to see if that was. I don't. I don't recall it [HPV vaccination] at all. So maybe it was something that I opted out of. And again, is that because there wasn't enough information given? And so on.’ (Patient 14; screened, unsure if vaccinated)*
	*‘I wonder if like antenatal clinics can do a little more actually. And midwives, because if they check the cervical screening history… that is the perfect opportunity to pick it up.’ (HCP 6)*
Theme 2: Structural, practical and place based barriers to access	
*Reduced access in primary care*	*‘Just ringing the doctors to book the appointment was a mission in itself.’ (Patient 2; screened, not vaccinated)*
	*‘Work… family, kids and you're putting everyone else first all the time… we're too busy looking after everyone else all the time, aren't we.’ (Patient 6; screened, vaccinated)*
	*‘By the time the appointment time has turned up, they tend to forget because it's three weeks advance.’ (HCP 5)*
*Place‐based and socioeconomic constraints*	*‘for somebody to come here for a cervical screen, it's you know, where we're going to park. Do I have enough money or for a taxi or whatever?’ (HCP 3)*
	*‘I prefer the GP, it's closer to my home. I can just walk it.’ (Patient 15; screened, vaccinated)*
	*‘They don't want to be seen walking into a sexual health clinic… Don't want people to think, oh, you know, why is she going in there?’ (HCP 3)*
*Competing roles and limited capacity for self‐care*	*‘You just think you could do it all. You could work, you could family, kids and you're putting everyone else first all the time.’ (Patient 6; screened, vaccinated)*
	*Screening considered to be ‘non‐urgent' unless symptomatic. ‘I think maybe before I had kids, it'd probably be one of my top priorities.’ (Patient 11; screened, vaccinated)*
Theme 3: Cultural and social contexts influencing engagement	
*Taboo and modesty*	*‘because the way we've all been brought up, well the majority of us you shouldn't really get it done before marriage.’ (Patient, 8)*
	*‘we get young women coming in that are not sexually active or they don't think they need a psychology test because they think that, you know, HPV is only sort of contracted when you've had sexual intercourse, that kind of thing.’ (HCP 1)*
	*Embarrassment discussing private parts, especially before marriage. ‘I was whispering, can I make a smear test please?’ (Patient 11; screened, vaccinated)*
	*Even talking about sex or your private parts is not necessarily something that's discussed.’ (HCP 3)*
*Influence of family, partners and community narratives*	*‘Some of them have to go back and get their husbands permission before they come back.’ (HCP 5)*
	*‘My mum was very pro screening… but my friend's mum was like no, don't go for it.’ (Patient 10; screened, vaccinated)*
	*‘They don't want to be seen walking into a sexual health clinic… Don't want people to think, oh, you know, why is she going in there?’ (HCP 3)*
*Trust, representation and cultural safety*	*I think the uptake has improved in the surgery, you know, because like I say, I can speak the language.’ (HCP 5)*
	*‘I think people who have had it done, if they talk about it more within communities, I think that's the only thing.’ (Patient 13; screened, unsure if vaccinated)*
	*‘We have to be careful about where we use these videos. It's quite embarrassing in the Asian community.’ (HCP 5)*
Theme 4: Emotional drivers of engagement and avoidance	
*Fear of pain, exposure and loss of dignity*	*‘I'm thinking of like big metal forceps… like they're going to open you up.’ (Patient 4; not screened, vaccinated)*
	*‘I was like I have to get my bits out in front of another person. That's what I think I was more nervous about’ (Patient 10; screened, vaccinated)*
	*‘I had just given birth so I was very traumatised from giving birth so I said no.’ (Patient 12; not screened, vaccinated)*
*Fear of results and diagnostic consequences*	*‘I was on edge, you know, for eight days. I was like, oh yeah, cancer, what would I do? Yeah, you know what I'm saying? So very scary.’ (Patient 5; screened, unsure if vaccinated)*
	*‘I just prevent myself from doing the test.’ (Patient 6; screened, vaccinated)*
*Reframing through positive experiences*	*‘The thought of the process is very traumatic… I'm a stranger and I'm looking at their private parts and touching them and that can bring back post‐traumatic stress disorder for them… So for me, I'm trying to establish what the issue is first…It will take a couple of appointments to establish that. It might be educating them… So I kind of do it gradually in to what suits them.’ (HCP 2)*
	*‘And it's not as bad as you think it's going to be.’ (Patient 4; not screened, vaccinated)*
Theme 5: Opportunities for change: Community led and culturally responsive	
*Tailoring intervention content and delivery to community contexts*	*‘But you know what I think personally, if there was a picture, honestly, and you said to put this inside you for not even 10 s… I would have said, actually, I would have done it.’ (Patient 5; screened, unsure if vaccinated)*
	*‘it looks like they've typed a letter from the top of the page to the bottom and all of them words. we're not reading it.’ (Patient 5)*
	*‘I've had so many ladies in floods of tears saying I don't understand what this letter means.’ (HCP 2)*
	*‘Maybe if a letter or like a video… maybe something should be brought up in a different language, like in their language like in Urdu or Punjabi or something… English isn't obviously their first language so maybe in another language.’ (HCP 6)*
	*I'm very passionate about education… And I think when they say ‘Oh my gosh, my friend had a speculum in and they opened her vagina that big' and I'm like, we would never open your vagina that big… and I'll say you know, it's not as bad as labour… and I'm going to talk you through it’ (HCP 2)*
	*‘I think people who have had it done, if they talk about it more within communities…’ (Patient 13; screened, unsure if vaccinated)*
	*‘explain with like talks… like how we have the coffee and chats…get all the South Asian women together in one rooms… just explain how it goes.’ (Patient 15; screened, vaccinated)*
*System‐level flexibility and partnership working*	*‘work. family, kids and you're putting everyone else first all the time… you just can't make time out for yourself, then so, you know, it's too late.’ (Patient 6; screened, vaccinated)*
	*‘They can't get anyone to look after the kids… it's just things like that.’ (HCP 5)*
	*‘GP appointments and nurse appointments are horrendous and I think they've only just got worse to be honest’ (Patient 13; screened, unsure if vaccinated)*
	*‘obviously there is more we could do, but it's down to things like staffing… it's having the funding.’ (HCP 1)*
	*‘We used to join forces with primary care and colposcopy, but that's' gone now. There's a lack of communication… it's unfortunate because it was really beneficial.’ (HCP 6)*
	*‘So a community videos for Asian women to get support with health issues they don't want to talk about with anybody else or whether they get judged and, you know, things like that. There's nothing, nothing at all.’ (HCP 4)*
	*‘If there were sessions at the community centre, like coffee and chats, where someone explained it properly, people would go.’ (HCP 5)*
	*‘I've not come across any, any cervical screening seminars or groups or you know in the local community centre. Definitely definitely [would be beneficial]. (Patient 6; screened, vaccinated)*

Participants proposed a range of strategies to improve engagement, emphasising the importance of accessible and culturally sound information. Visual explanations were especially valued, as highlighted by one participant, ‘you know what I think personally, if there was a picture, honestly, and you said to put this inside you for not even 10 s… I would have said, actually, I would have done it.' (Patient, 5). On the other hand, standard written communication was often perceived as inaccessible, with one participant noting, ‘it looks like they've typed a letter from the top of the page to the bottom and all of them words. we're not reading it' (Patient, 5).

Multilingual resources were considered essential for inclusivity: ‘Maybe if a letter or like a video… maybe something should be brought up in a different language, like in their language like in Urdu or Punjabi or something.' (HCP, 6). Additionally, honest explanations of discomfort were also considered to be helpful: ‘I'm very passionate about education… and I'll say you know, it's not as bad as labour… and I'm going to talk you through it' (HCP 2).

Participants also highlighted the value of peer dialogue and community‐based engagement, suggesting informal group discussions as a supportive learning environment, ‘explain with like talks… like how we have the coffee and chats…get all the South Asian women together in one room… just explain how it goes.' (Patient, 15). The lack of perceived absence of local support was also noted, with one HCP suggesting, ‘a community link for Asian women to get support with health issues they don't want to talk about with anybody else… there's nothing, nothing at all' (HCP, 4).

System‐level flexibility was also identified as necessary to support attendance. Women described persistent challenges balancing screening with caregiving responsibilities, while HCPs emphasised resource constraints, noting that expanding services was often ‘down to things like staffing' and ‘having the funding.' (HCP, 1).

## 4 Discussion

### 4.1 Summary

This study explored barriers and facilitators to cervical screening participation among South Asian women aged 25–29 in Blackburn with Darwen, alongside healthcare professionals' perspectives on screening delivery. By examining both patient and provider experiences within a single locality, the findings offer context‐specific insight into how knowledge, emotional responses, cultural norms, and structural constraints intersect to shape cervical screening behaviour.

Our findings demonstrate that screening participation among South Asian women aged 25–29 is shaped by multiple, interrelated factors. Limited understanding and misconceptions about the purpose and procedure of cervical screening reduced perceived need for screening, while fear, embarrassment, and concerns about community judgement shaped decision‐making and led some women to delay or avoid screening. Cultural norms related to modesty and sexuality, expectations associated with marriage status, and concerns around visibility within close‐knit communities were identified as specific barriers. Practical challenges, including difficulties accessing appointments and competing responsibilities, further constrained participation. Healthcare professionals described adapting their approach to support women but highlighted ongoing pressures related to staffing and service capacity [23]. Notably, the findings also revealed missed opportunities to promote understanding of cervical screening during adolescence, despite most participants reporting HPV vaccination. Rather than suggesting that vaccination and screening are competing approaches, the findings highlight the importance of linking messages about HPV vaccination with clear information about the continuing role of cervical screening. This may be of particular relevance for younger South Asian women entering the screening programme following adolescent vaccination.

Furthermore, it is also important to consider participants' screening histories when interpreting these findings. Ten of the fifteen women had attended cervical screening on at least once occasion prior to interview, with the remainder describing delayed attendance or non‐attendance. Women who had previously attended, even if inconsistently, may differ in relevant ways from those who had never attended and thus the findings should be interpreted with this in mind. At the same time, participants' accounts provide valuable insight into the circumstances that supported behaviour from reluctance to attendance. Encouragement from family members and peers, increased openness to screening following marriage and reassurance gained from positive healthcare experiences were each described as helping women to overcome initial hesitancy. This suggests that barriers and facilitators to screening are not static, and that the point at which women transition from non‐attendance towards engagement may represent a valuable opportunity for intervention.

### 4.2 Comparison With Existing Literature

A key novel finding of this study was that knowledge gaps appear to be reinforced by missed educational opportunities across the life course, with several participants mentioning how cervical health was never discussed at school or college. The absence of early education may therefore allow misconceptions to persist into adulthood and shape future health decisions. These findings are especially relevant to younger South Asian women aged 25‐29, who represent a population increasingly entering the cervical screening programme following eligibility for HPV vaccination during adolescence. In the present study, 10 of the 15 women reported receiving the HPV vaccination, yet several participants described limited understanding of HPV and the continued importance of cervical screening. This provides an important distinction from research exploring vaccination and screening as separate preventive behaviours. Rather, the findings suggest that the transition from adolescent vaccination to adult cervical screening may represent an important point at which knowledge and understanding need to be reinforced. Previous research has identified an association between HPV vaccination and subsequent cervical screening attendance [7, 8], while evidence also indicates persistent inequalities in vaccination uptake and screening participation among some ethnic minority groups [5, 6]. Our findings add a qualitative perspective by illustrating how vaccination does not necessarily translate into a clear understanding of the purpose and continuing importance of cervical screening among younger South Asian women,

Consistent with existing literature, low awareness and knowledge remain key obstacles to cervical screening engagement among South Asian women [10, 13, 14, 16]. Reduced health literacy among minority ethnic groups has been linked to language barriers, differing health beliefs, and educational inequalities, all of which reduce an individual's ability to access and interpret health information [24]. Survey data from Jo's Cervical Cancer Trust further supports these findings, indicating that minority ethnic women are less likely to attend screening and that improved knowledge may increase participation [25]. The present study extends this evidence by focusing specifically on younger South Asian women approaching or engaging with the screening programme following adolescent HPV vaccination, highlighting the importance of connecting messages about vaccination with clear information about subsequent screening. This study further adds nuance by showing how culturally sensitive topics are frequently avoided in both formal and informal settings, reinforcing knowledge gaps.

Cultural beliefs linking screening to marriage, childbirth, or sexual activity, alongside assumptions that the absence of symptoms equates to the absence of risk, were prominent. These culturally shaped misunderstandings further led to reduced screening attendance. These findings align with prior research highlighting the role of modesty, virginity, and marital status in shaping screening attitudes [10, 11, 15]. Fear of community judgement and concerns about visibility when accessing services mirrored findings among British Pakistani and Bangladeshi women [16]. Importantly, this study demonstrated intra‐community variation, with family members acting as both facilitators and barriers, challenging homogenising narratives about South Asian culture.

Alternative screening modalities may help address some identified barriers. Whitley et al. [26] suggest that self‐sampling methods, including urine testing and vaginal self‐swabs, are acceptable among ethnically diverse women in North West England due to increased privacy, comfort and control over the screening process. These findings are particularly relevant given the emphasis on modesty and embarrassment observed in this study.

Emotional responses, including fear of pain, exposure, and potential diagnosis, were central to screening avoidance, resonating with prior research [10, 11, 13, 15]. Healthcare professionals described adapting their language to reduce anxiety, for example by avoiding explicit references to cancer. While this approach may reduce anxiety, it may also limit opportunities for transparent discussion about cancer prevention and risk, highlighting a tension between reassurance and informed decision‐making. Positive screening experiences, however, had the potential to reframe expectations and encourage peer advocacy, suggesting that experiential knowledge within communities may be a powerful facilitator.

Overall, these findings distinguish barriers that appear broadly generalisable across underscreened populations from those that are more specific to or more acutely experienced by South Asian women. Structural and practical barriers, including inflexible appointment systems, competing caring responsibilities and constrained health literacy, echo barriers reported among other underscreened groups, including migrant and low‐income populations [13, 15, 27]. In contrast, taboo linked to marital and sexual status, family and community and fear of reputational harm within close‐knit communities emerged as more distinctly important within this study. This distinction bears practical significance, as generic interventions e.g. reminder letters or population‐level campaigns, are, in isolation, unlikely to address barriers that are culturally embedded. Rather, our findings highlight the need for community‐embedded, culturally responsive approaches, delivered through trusted community spaces, access to female practitioners, multilingual resources and opportunities for confidential discussion away from family. Together, this may represent the most meaningful route to improving screening engagement among South Asian women.

### 4.3 Strengths and Limitations

To our knowledge, this is the first qualitative study to explore cervical screening barriers among South Asian women aged 25–29, a cohort likely eligible for HPV vaccination following its introduction in England in 2008. A key strength is the inclusion of both women's and healthcare professionals' perspectives, offering a more comprehensive understanding of barriers and highlighting opportunities for intervention, particularly in relation to knowledge gaps.

However, the study took place in a single geographical area characterised by high ethnic diversity and low screening uptake, which may limit generalisability beyond similar settings. The absence of routinely collected, individual‐level ethnicity data within NHS screening statistics further restricts the ability to contextualise findings within wider epidemiological patterns, despite persistent place‐based evidence of inequality. Nevertheless, the findings are likely informative for other areas in England with comparable sociodemographic characteristics.

Several additional limitations should be noted. Recruitment relied on opportunistic and snowball sampling, which may have resulted in sampling and selection bias, as participants willing to take part may have been more engaged or motivated than women entirely disengaged from screening. However, a third of the study participants have either not taken part in screening or had delayed participation, which mirrors cervical screening participation patterns in Blackburn with Darwen.

HCPs were recruited based on their roles delivering cervical screening and promoting participation among non‐responders in Blackburn with Darwen; however, no data were collected regarding their years of experience and the practice characteristics (e.g. coverage rates among target population). These may have influenced their perspectives on screening barriers and facilitators among young South Asian women aged 25–29 in Blackburn with Darwen.

All interviews were conducted in English, excluding non‐English‐speaking women; given that language barriers were identified as an important influence on screening engagement, this may have led to underestimation of their impact. Screening and HPV vaccination status were self‐reported, and some participants were uncertain about their vaccination history, limiting the ability to draw firm conclusions regarding the relationship between HPV vaccination and screening behaviour. However, this pattern could also point to a gap in HPV‐related understanding among vaccinated participants rather than a demonstrated causal relationship between vaccination and screening behaviour. Future research would benefit from the inclusion of interviews conducted in community languages and triangulation with health records where feasible.

### 4.4 Implications for Research and Practice

The findings of this research have important implications for primary care and public health practice. Culturally responsive communication strategies are needed, including proactive discussions addressing misconceptions about both cervical screening and the HPV vaccine. Participants highlighted practical improvements such as clearer communication, multilingual resources, access to female practitioners, and delivery of information within trusted community spaces, aligning with existing recommendations to improve screening uptake among minority ethnic populations [12, 13, 28].

While national campaigns such as ‘help us help you – cancer screening saves lives' aim to improve awareness and encourage attendance [29], reminders alone appear insufficient where psychological capabilities and physical opportunities are limited. Persistent barriers are likely to be reduced through flexible appointment systems, tailored information, improved navigation, and partnership working with voluntary and community organisations. Expanding access to self‐sampling and delivering support beyond traditional primary care settings may be particularly beneficial for younger South Asian women. Further research is needed to understand how such approaches can be effectively implemented and sustained.

## 5 Conclusion

In Blackburn with Darwen, cervical screening behaviour among young South Asian women aged 25‐29 is shaped by intersecting knowledge‐based social, cultural, and structural factors. Addressing inequalities in screening uptake therefore requires a multifaceted approach incorporating culturally responsive communication, flexible service design, and strong community partnership. Without coordinated action across these domains, existing disparities in cervical screening participation are likely to persist.

## Author Contributions

**Katie Merry:** conceptualisation, methodology, data curation, writing – review and editing. **Yasemin Hirst:** conceptualisation, methodology, supervision, formal analysis, writing – original draft, writing – review and editing. **Rebecca Jefferson:** supervision, writing – original draft, writing – review and editing. **Zainab Laheri:** formal analysis, writing – original draft, writing – review and editing. **Sarah Johns:** methodology, writing – review and editing. All authors read and approved the final manuscript.

## Ethics Statement

Ethical approval was granted by the University of Lancashire's (UCLan) HEALTH ethics committee: HEALTH 01169. Participants received an information sheet and provided informed written consent before participation.

## Conflicts of Interest

The authors declare no conflicts of interest.

## Supporting information


Supporting File 1

Supporting File 2

Supporting File 3

## Data Availability

Due to the sensitive and potentially identifiable nature of the qualitative data and the conditions of ethical approval, the datasets are not publicly available. Anonymised data may be available from the corresponding author upon reasonable request.

## References

[1] NHS England . 2025a. *Cervical cancer elimination by 2040 – plan for England*. [Online]. NHS England. Last Updated: 2025, accessed 15 February 2026, https://www.england.nhs.uk/long-read/cervical-cancer-elimination-by-2040-plan-for-england/#:~:text=T.

[2] NHS England . 2023. *HPV Vaccine*. [Online]. NHS England. Last Updated: 2023, accessed 15 February 2026, https://www.nhs.uk/vaccinations/hpv-vaccine/#:~:text=Who%20should%20have%20the%20HPV,free%20on%20the.

[3] NHS England . 2025b. *Topic 1: the NHS Cervical Screening Programme (NHSCSP)*. [Online]. NHS England. Last Updated: 2025, accessed 15 February 2026, https://www.gov.uk/government/publications/cervical-screening-cervical-sample-taker-training/topic-1.

[4] M. Falcaro , A. Castañón , B. Ndlela , and P. Sasieni , “Association Between HPV Vaccination and Cervical Screening Policy Changes and Cervical Cancer Incidence and grade‐3 Cervical Intraepithelial Neoplasia Incidence in England, 2006–2020: a Population‐Based Trends Analysis,” Lancet Regional Health. Europe 49 (2025): 101157, 10.1016/j.lanepe.2024.101157. PMC11697118 39759575

[5] D. Chilekwa 2022, A Cross‐Sectional Analysis Of Ethnic Inequalities In Cervical Screening Uptake In The Uk Using Understanding Society. Conference Paper.

[6] E. Dema , R. Osman , K. Soldan , N. Field , and P. Sonnenberg , “Are There Any Sociodemographic Factors Associated With Non‐Uptake of HPV Vaccination of Girls in High‐Income Countries With School‐Based Vaccination Programmes? A Systematic Review,” Journal of Epidemiology and Community Health 79 (2025): 388–396. 10.1136/jech-2024-222488 PMC12015032 39715632

[7] H. Beer , S. Hibbitts , S. Brophy , M. A. Rahman , J. Waller , and S. Paranjothy , “Does the Hpv Vaccination Programme Have Implications for Cervical Screening Programmes in the UK?,” Vaccine 32, no. 16 (2014): 1828–1833, 10.1016/j.vaccine.2014.01.08. PMC3991313 24530938

[8] S. Shpendi , P. Norman , J. Gibson‐Miller , and R. Webster , “Cervical Screening Attendance in Young Women and People With a Cervix: An Application of the Com‐B Model,” British Journal of Health Psychology 30, no. 3 (2025): 1–14, 10.1111/bjhp.70016. PMC12361587 40826314

[9] H. C. Johnson , E. I. Lafferty , R. M. Eggo , et al., “Effect of Hpv Vaccination and Cervical Cancer Screening in England by Ethnicity: A Modelling Study,” Lancet Public Health 3, no. 1 (2017): e44–e51, [Online], 10.1016/S2468-2667(17)30238-4. PMC5765530 29307388

[10] Z. Alam , H. Deol , J. A. Dean , and M. Janda , “Reasons Behind Low Cervical Screening Uptake Among South Asian Immigrant Women: A Qualitative Exploration,” International Journal of Environmental Research and Public Health 19, no. 3 (2022): 1527. 10.3390/ijerph19031527 PMC8834789 35162550

[11] K. Devotta , A. Lofters , J. Bender , and P. O'Campo , “Perceptions of Cervical Screening Uptake Amongst South Asian Women in Ontario, Canada: A Concept Mapping Study,” BMC Public Health 25 (2025): 265. 10.1186/s12889-025-21448-6 PMC11752636 39844167

[12] M. Haith‐Cooper , D. Payne , N. Almas , M. Rattray , and H. Iqbal , “Supporting Cancer Screening Uptake in South Asian Women: The Wise Up to Cancer Public Health Intervention,” Taylor & Francis Online 35, no. 1 (2025), 10.1080/09581596.2025.2520406.

[13] L. A. V. Marlow , J. Waller , and J. Wardle , “Barriers to Cervical Cancer Screening Among Ethnic Minority Women: A Qualitative Study,” Journal of Family Planning and Reproductive Health Care 41, no. 4 (2015): 248–254. 10.1136/jfprhc-2014-101082 PMC4621371 25583124

[14] R. Sokal , “A Critical Review of the Literature on the Uptake of Cervical and Breast Screening in British South Asian Women,” Quality in Primary Care 18, no. 4 (2010): 251–261. 20836941

[15] O. A. Bolarinwa and N. Holt , “Barriers to Breast and Cervical Cancer Screening Uptake Among Black, Asian, and Minority Ethnic Women in the United Kingdom,” BMC Health Services Research 23, no. 390 (2023): 390. 10.1186/s12913-023-09410-x PMC10122823 37087506

[16] R. M. A. de Cuevas , P. Saini , D. Roberts , et al., “A Systematic Review of Barriers and Enablers to South Asian Women's Attendance for Asymptomatic Screening of Breast An,” BMJ Open 8, no. 7 (2018): e020892, 10.1136/bmjopen-2017-020892. PMC6042536 29982210

[17] Care Quality Commission , 2025, *Blackburn With Darwen Borough Council: Local Authority Assessment*. [Online]. Care Quality Commission. Last Updated: 2025, accessed 20th February 2026, https://www.cqc.org.uk/care-services/local-authority-assessment-reports/blackburndarwen-0725.

[18] Healthwatch, Blackburn with Darwen . 2025. *Improving Access and Awareness in Cervical Screening in Blackburn with Darwen*. [Online]. Healthwatch, Blackburn With Darwen. Last Updated: 2025, accessed 16th February 2026, https://nds.healthwatch.co.uk/sites/default/files/.

[19] A. Tong , P. Sainsbury , and J. Craig , “Consolidated Criteria for Reporting Qualitative Research (COREQ): A 32‐item Checklist for Interviews and Focus Groups,” International Journal for Quality in Health Care 19, no. 6 (2007): 349–357. 10.1093/intqhc/mzm042 17872937

[20] S. J. Howcutt , P. Saini , C. Henshall , J. Brett , E. Watson , and L. A. Smith 2022. “Visualizing Community Networks to Recruit South Asian Participants for Interviews About Bowel Cancer Screening,” Journal of Cancer Policy 32: 100333, 10.1016/j.jcpo.2022.100333. 35560264

[21] S. J. Roberts , M. A. Saylor , S. Ivynian , and M. DiGiacomo , “Data Saturation and Information Power: Sample Size in Qualitative Research—When Is Enough and How to Assess?,” European Journal of Cardiovascular Nursing 25, no. 5 (2026): 1069–1074. 10.1093/eurjcn/zvag046 41693424

[22] V. Braun and V. Clarke , Thematic Analysis: A Practical Guide (Sage, 2021).

[23] T. Powell , C. Baker , and D. Foster (2024). Capacity Pressures In Health And Social Care In England. [Online]. UK Parliament: House of Commons Library. Last Updated: 2024, accessed February 27, 2026, https://commonslibrary.parliament.uk/capacity-pressures-in-health-andsocial-care-in-england/#:~:tex.

[24] A. Waqar , “From Awareness to Action: Tackling Health Inequalities Through Improved Health Literacy Among Ethnic Minorities in Engla,” Electronic Journal of General Medicine 22, no. 4 (2025): em660.

[25] T. Thomas 2020, *Cervical Cancer: Minority Ethnic Women More Likely To Miss Screenings In Pandemic*. [Online]. The Guardian. Last Updated: 2020, accessed 26 February 2026, https://www.theguardian.com/society/2020/aug/25/cervical-cancer-bame-women-more-likely-to-miss-scree.

[26] S. Whitley , R. L. Hawkins , J. C. Davies , et al., “Acceptability of Self‐Sampling for Cervical Screening in Ethnically Diverse Groups in Northwest England: A Focus Group,” Health Expectations 28, no. 46 (2025): e70338, [Online], 10.1111/hex.70338. PMC12312516 40742376

[27] M. Jackowska , C. von Wagner , J. Wardle , D. Juszczyk , A. Luszczynska , and J. Waller , “Cervical Screening Among Migrant Women: A Qualitative Study of Polish, Slovak and Romanian Women in London, UK,” Journal of Family Planning and Reproductive Health Care 38, no. 4 (2012): 229–238. 10.1136/jfprhc-2011-100144 PMC3970719 22219504

[28] V. Kiridaran , M. Chawla , and J. V. Bailey , “Views, Attitudes and Experiences of South Asian Women Concerning Sexual Health Services in the Uk: A Qualitative Study,” European Journal of Contraception & Reproductive Health Care: The Official Journal of the European Society of Contraception 27, no. 5 (2022): 418–423. 10.1080/13625187.2022.2096216 35899823

[29] Department of Health & Social Care 2022, Cervical Screening. [Online]. Department of Health & Social Care, accessed 27 February 2026, https://campaignresources.dhsc.gov.uk/campaigns/help-us-help-you-cancer/cervical-screening/.

