# Supplementary material for: Understanding Cervical Screening Engagement Among Young South Asian Women: Perspectives of Women and Healthcare Professionals
Source: Health Expect. 2026 Nov 5;29(6):e70960. doi: 10.1111/hex.70960 (PMC13735218; doi:10.1111/hex.70960)
Supplement: Supplementary file 2 [file HEX-29-e70960-s001.docx]

**Supplementary Material B - Cervical screening questions for Healthcare Professionals version 1.0, dated 28.08.2024**

*Research Question: Understanding the low uptake of cervical screening in young women aged 25- 29 from South Asian backgrounds in Blackburn with Darwen*

Hello, my name is XXX and am currently doing a research internship with the NIHR to understand the low uptake of cervical screening amongst young South Asian women between the ages of 25-29. The purpose of this interview is to explore your experience and views of cervical screening and how improvements can be implemented where needed. We would like to encourage you to go into as much detail as you can, what you have to say is very valuable information. Our interview/chat will last approximately 30 minutes. Everything you say is strictly confidential and anonymous. There are no right or wrong answers; I simply want to hear your views. I am not here to judge your comments but rather to collect them. Quotes may be used when the results of the study are being presented or published, but again you will not be personally identified.

**Job title:**

**What is your knowledge and understanding of cervical screening?**

**e.g. ask elaborate on coverage and inequalities at their practice if possible**

**Patient Concerns**

**What do you think the most common concerns or misconceptions patients have about cervical screening?**

*Prompts: Do patients express any negative emotions towards screening? If yes, can you please elaborate?*

**Can you describe the steps you take during a cervical screening procedure to ensure patient comfort and accuracy of the test?**

*Prompts: Do you offer additional support to patients who have worries or concerns?*

**How do you support patients if they are less inclined to attend their appointments or do not attend screening?**

*Prompt: Longer appointment times, chaperones, trial run appointments, educational sessions?*

**What training do you receive on cervical screening techniques and guidelines?**

*Prompt: How do you stay updated on the latest research and recommendations in cervical cancer screening?*

**Cultural and Social Barriers**

**How do you feel cultural or social factors affect women's willingness to participate in cervical screening?**

*Prompt: Embarrassment, Taboo, stigma or fear of judgment*

**What changes or improvements do you think can be made for the specific communities and demographics that show lower participation rates?**

**Accessibility Issues**

**What logistical barriers do you feel patients face in accessing cervical screening services?**

**Prompt: (e.g., location, timing, transportation and childcare)**

**Educational strategies**

**What educational materials or methods do you feel have proven most effective in informing patients about the importance of cervical screening?**

*Prompt: Information leaflets in different languages/ easy read?*

**How can healthcare professionals better communicate the benefits and importance of regular cervical screening?**

**How can healthcare professionals collaborate with community organisations to raise awareness and encourage screening?**
