# Supplementary material for: Understanding Cervical Screening Engagement Among Young South Asian Women: Perspectives of Women and Healthcare Professionals
Source: Health Expect. 2026 Nov 5;29(6):e70960. doi: 10.1111/hex.70960 (PMC13735218; doi:10.1111/hex.70960)
Supplement: Supplementary file 3 [file HEX-29-e70960-s003.docx]

**Supplementary Material A - Cervical screening Interview questions**

**Research Question: Understanding the low uptake of cervical screening in young women aged 25- 29 from South Asian backgrounds in Blackburn with Darwen.**

Hello, my name is xxx and am currently doing a research internship with the NIHR to understand the low uptake of cervical screening amongst young South Asian women between the ages of 25-29. The purpose of this interview is to explore your experience and views of cervical screening and how improvements can be implemented where needed. We would like to encourage you to go into as much detail as you can, what you have to say is very valuable information. Our interview/chat will last approximately 30 minutes. Everything you say is strictly confidential and anonymous. There are no right or wrong answers; I simply want to hear your views. I am not here to judge your comments but rather to collect them. Quotes may be used when the results of the study are being presented or published, but again you will not be personally identified.

**Are you happy for me to record this interview for note taking purposes?**

If Yes – Press record

If No, I will take notes

**Would you be able to confirm your consent to take part in the study?**

Yes – Continue

No-Terminate the interview

**About you**

**If you are happy to share, would you please tell us your age, and ethnicity for our records?**

Age:

Ethnicity:

Disability: Yes/No

**1.Can you explain to me based on what you currently know, what do you think causes cervical cancer?**

Prompts-

Do you know how the test for cervical cancer is carried out?

Do you know how they take the test samples?

**2.Have you ever been for a cervical screening?**

Yes

No Q3 & Q4

**If yes can you tell me about your experience of cervical screening?**

Prompts-

*How was your experience attending the cervical screening?*

*Did you have any concerns or worries? Could you tell me a bit more about it?*

*Is there improvements which could be made to make you consider going back if a previous experience put you off?*

**3.If no, please tell me about your decision to not attend the screening?**

Prompts:

*Was there a specific reason which made you decide?*

*What were your concerns?*

**4.What would make you more likely to attend in the future?**

**5.What did you think when you first received the invitation?**

Prompts:

*How did it make you feel?*

*Did you seek any information about cervical screening?*

*How were you contacted?*

*Was the letter easy for you to understand? Was there enough information to make a decision to book your appointment?*

**6.How did you find booking your appointment?**

*Prompt:*

*Was it accessible to you? Place of choice- opening times, flexible, travel, childcare etc*

*Was it at your GP? Were you happy with having it here? Or would you prefer it somewhere else? If yes, where?*

**7.Would you ever talk to your friends or family about cervical screening?**

**8.What do you think could be done to encourage more South Asian women to take up cervical screening?**

*Do you feel there is a lack of awareness?*

*What do you feel can be done to improve this?*

**Lets now talk about the HPV vaccine (Human papillomavirus vaccine)**

**9.Have you ever been offered the HPV vaccine?**

**Yes /No**

**10.Have you had the vaccine?**

**Yes/No**

**If yes, was this when you were at school or by your GP or other healthcare professional?**

**If not, what were your reasons why?**

**End of interview, thank you for your time today. Is there anything you would like to add before I stop recording?**

**Reminder of support links, withdrawal process.**
